# Assessment of RNAlater^®^ as a Potential Method to Preserve Bovine Muscle Proteins Compared with Dry Ice in a Proteomic Study

**DOI:** 10.3390/foods8020060

**Published:** 2019-02-05

**Authors:** Yao Zhu, Anne Maria Mullen, Dilip K. Rai, Alan L. Kelly, David Sheehan, Jamie Cafferky, Ruth M. Hamill

**Affiliations:** 1 Teagasc Food Research Centre, Ashtown, Dublin D15KN3K, Ireland; yao.zhu@teagasc.ie (Y.Z.); Anne.mullen@teagasc.ie (A.M.M.); dilip.rai@teagasc.ie (D.K.R.); jamie.cafferky@teagasc.ie (J.C.); 2 School of Food and Nutritional Sciences, University College Cork, Cork T12 K8AF, Ireland; a.kelly@ucc.ie; 3 Department of Chemistry, Khalifa University, Abu Dhabi PO Box 127788, UAE; david.sheehan@ku.ac.ae

**Keywords:** muscle proteins, one-dimensional electrophoresis, bovine proteomics, LC-MS/MS, sample preparation

## Abstract

RNAlater^®^ is regarded as a potential preservation method for proteins, while its effect on bovine muscle proteins has rarely been evaluated. Bovine muscle protein samples (*n* = 12) collected from three tender (Warner–Bratzler shear force: 30.02–31.74 N) and three tough (Warner–Bratzler shear force: 54.12–66.25 N) Longissimus thoracis et lumborum (LTL) samples, preserved using two different sampling preservation methods (RNAlater^®^ and dry ice), at two *post mortem* time points (day 0 and day 14), were characterized using one-dimensional electrophoresis. Fourteen bands with molecular weights ranging from 15 to 250 kDa were verified, both in the dry ice and RNAlater^®^ storage groups, at each time point, using image analysis. A shift from high to low molecular weight fragments, between day 0 and day 14, indicated proteolysis of the muscle proteins during post mortem storage. Liquid chromatography-tandem mass spectrometry (LC-MS/MS) analyses and database searching resulted in the identification of 10 proteins in four bands. Protein profiles of muscle preserved in RNAlater^®^ were similar to those of muscle frozen on dry ice storage, both at day 0 and day 14. The results demonstrate that RNAlater^®^ could be a simple and efficient way to preserve bovine muscle proteins for bovine muscle proteomic studies.

## 1. Introduction

Meat proteomics research critically depends on the reliability of tissue samples. It is essential to avoid proteolysis and enzymatic activity in sample preservation, so as to conserve the structural integrity of muscle proteins. Snap freezing of samples in liquid nitrogen, at −196 °C, or on dry ice, at −79 °C, is an efficient method to stabilize samples. Nonetheless, they can be difficult to carry out in-factory. RNAlater^®^, an aqueous solution, is extensively used to preserve RNA in fresh tissue and cell samples for clinical genomic and transcriptomic studies [1]. The main compounds in RNAlater^®^ are quaternary ammonium sulphates and cesium sulphate, which denature proteins, including RNases, DNases and proteases, thereby stabilizing RNA, DNA and protein content [2]. 

RNAlater^®^ has been investigated as an effective potential preservation method for proteins originating from bacteria, plants, blood vessels and human colon mucosal biopsies [2,3,4]. However, comparative proteomic studies between frozen and RNAlater^®^ preserved tissues have shown some differences across tissues, for example, more proteins were detected in mouse leukocytes preserved using RNAlater^®^ [5], compared to frozen tissue, and three of 20 analysed proteins were more abundant in snap-frozen mouse liver [6], whereas one was more abundant in RNAlater^®^ preserved tissue. Furthermore, it was observed that 84 soluble proteins and 120 membrane-bound proteins were expressed differently in RNAlater^®^ fixed samples compared with liquid nitrogen [7].

To date, little attention has been paid to the impact of RNAlater^®^ on proteomic profiles from muscle tissue. In a study on gulf killifish preserved in liquid nitrogen and RNAlater^®^, it was demonstrated that, in contrast to their findings in other tissues, for skeletal muscle and brain, snap freezing and preservation in RNAlater^®^ showed similar protein abundances [8]. Large biobanks of samples stored in RNAlater^®^ exist for muscle transcriptomics, which could serve as an important resource for meat proteomics if muscle protein abundance profiles post mortem were similar to those in snap frozen muscle. However, no studies are available which assess the potential differential effect of storage in RNAlater^®^ versus freezing on dry ice on post mortem proteolytic patterns in bovine muscle.

The objective of this study was to compare proteome patterns of *post mortem* bovine muscle stored in RNAlater^®^ or dry ice by using one-dimensional sodium dodecyl sulphate-polyacrylamide gel electrophoresis (1D SDS-PAGE). To the best of our knowledge, this is the first systematic study of the effect of RNAlater^®^ on bovine muscle proteins, with a view to investigate its potential as a specimen-stabilization solvent for beef proteomics.

## 2. Materials and Methods

### 2.1. Tissue Sampling

Twenty-eight Limousin bulls were slaughtered in an EU-licensed abattoir under standard conditions. *Longissimus thoracis et lumborum* (LTL) samples (2 g) were collected approximately 1 h post-slaughter (day 0), from the region of the 10th rib of each carcass on the right side, in the chill room. Muscle (LTL) tissue samples (1 g) were finely macerated and stored at 4 °C in 5 mL RNAlater^®^ for 24 h and, then, RNAlater^®^ was removed by plastic pipette and the sample was transferred to −80 °C. In parallel, 1 g tissue was frozen on dry ice, and transferred to a freezer at −80 °C for downstream analysis. Two steaks (2.54 cm thick) from each LTL was excised on day 2 post mortem, vacuum packaged and stored at 4 °C, and one was frozen at day 14 for shear force analysis. The other was sampled for proteomics, applying the same preservation methods as on day 0. 

### 2.2. Warner–Bratzler Shear Force Measurement

Steaks from each of the 28 animals were subjected to Warner–Bratzler shear force in accordance with American Meat Science Association (AMSA) guidelines [9]. Steaks (2.5 cm) were thawed in a circulating water bath at 20 °C, cooked in a circulating water bath (Grant Instruments Ltd., Cambridge, UK) at 72 °C, until an internal temperature of 69 °C was achieved. After cooking, the samples were stored at 4 °C overnight. 

Eight cores (1.25 cm diameter) were taken from each steak parallel to fibre direction. Cores were sheared at the central point on a Warner–Bratzler device, attached to an Instron Universal testing machine with a 50 mm/min crosshead speed. Highest and lowest shear values were excluded for each sample and the mean values for 6 cores were reported. 

### 2.3. Extraction of Muscle Proteins

The three most tender and three toughest animals, based on the shear force values (Section 2.2), were selected for proteomic analysis. All tissue samples (100 mg), RNAlater®-preserved and dry ice-preserved, at day 0 and day 14, from each of these six animals (*n* = 24 samples in total), were homogenized in 1 mL of 8.3 M urea, 2 M thiourea, 1% Dithiothreitol, 2% 3-[(3-cholamidopropyl) dimethylammonio]-1-propanesulfonate and 2% Immobilized pH gradient (IPG) buffer pH 3–10 (GE Healthcare). Homogenates were incubated with shaking for 30 min on ice, followed by a 30 min centrifugation at 10,000× *g* in order to remove unextracted cellular components, high molecular weight protein complexes and insoluble proteins. Protein concentrations of the supernatant were analysed at 595 nm using a microplate reader (BGM LABTECH, Ortenberg, Germany) based on the method of Bradford [10]. Bovine serum albumin (BSA) was used as the standard and the concentration of protein was shown to be between 3 to 6 mg/mL.

### 2.4. Proteomic Analysis

Protein extracts were separated by 1D SDS-PAGE [11] on commercial Mini-PROTEAN^®^ TGX™ precast gradient gels of 8.6 × 6.7 × 0.1 cm and 4%–20% polyacrylamide (Bio-Rad Laboratories, Deeside, UK). The tissue sample protein lysis liquid was mixed 1:1 with Laemmli sample buffer (Bio-Rad Laboratories, Deeside, UK). Twenty µL (5 µg protein) was loaded in each gel lane. Each of the 24 samples was loaded in duplicate in contiguous lanes over 6 gels. Ten µL of Precision Plus Protein™ (Bio-Rad Laboratories, Deeside, UK) was included as a standard in one lane per gel. All gels were further replicated giving a total of four lanes per sample (96 lanes) with each sample run on two separate gels. Gels were run under constant voltage at 90 V for 45 min, then at 120 V for 50 min. Subsequently, gels were stained with 50 mL Coomassie stain (Bio-Rad Laboratories, Deeside, UK) with gentle shaking for 1 h and then destained with distilled water.

### 2.5. Image Analysis

Gel images were acquired using a GS-800 densitometer (Bio-Rad Laboratories, Deeside, UK) and analysed by Quantity One software (Bio-Rad). Bands were detected by optical density and quantified by integrating the area under the curve of pixel intensity and band width (trace quantity × mm). For each sample, each of the four replicate lanes were scanned and averaged to give the sample mean abundance for each animal × treatment × time point. Statistical analysis of the sample mean abundance was undertaken using Genstat (Release 14.1, VSN international, London, UK). The analysis was a 2 × 2 × 2 factorial with terms for Quality (Tender, Tough), Treatment (Dry Ice, RNAlater^®^) and Time point (Day 0/14). Tukey’s test was used to compare means. A value of *p* < 0.05 was considered statistically significant.

### 2.6. Mass Spectrometry Analysis

Gels were transferred on to a glass plate and the protein bands of interest were excised with a sterile scalpel. Then, the gel pieces were spun down on a bench-top vortex. Bands were subjected to liquid chromatography-mass spectrometry at a core facility (King’s College, Aberdeen, UK) according to the method of Wilm [12].

## 3. Results and Discussion

**Figure 1:**
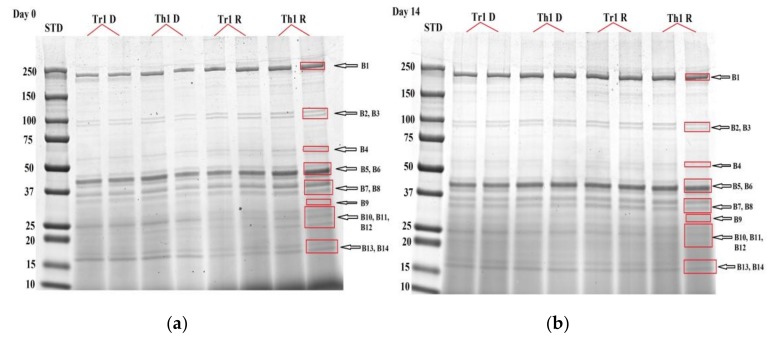
Four replicate lanes were run for each sample across 12 gels. Typical Coomassie-stained one-dimensional sodium dodecyl sulphate-polyacrylamide gel electrophoresis (1D SDS-PAGE) gels from the study, illustrating treatment, time point and quality samples run in duplicate, here data from tender sample 1 (Tr1) and tough sample 1 (Th1) are shown. The images above show proteins from *Longissimus thoracis et lumborum* (LTL) tender sample 1 in dry ice (Tr1 D) and RNAlater^®^ (Tr1 R) and tough sample 1 in dry ice (Th1 D) and RNAlater^®^ (Th1 R), at time point day 0 (**a**) and day 14 (**b**). Protein Plus protein standards (Bio-Rad Laboratories) comprising a wide range of molecular weights (10–250 kDa) were included in every gel for molecular weight determination (STD). The bands, as defined for quantitative image analysis are indicated with arrows (e.g., B1–B14).

As shown in Figure 1, 14 bovine muscle protein bands with molecular weights ranging from 250 kDa to 15 kDa (B1-B14) were detected using quantitative image analysis. Fourteen bands were detected in each lane for both RNAlater^®^-preserved and dry ice-preserved samples. 

No significant difference in optical density was detected for any band between the protein profiles of muscle preserved in RNAlater^®^ and those of muscle samples frozen on dry ice, both at day 0 and day 14 (Table 1). These results are consistent with findings of Abbaraju [7], which showed that RNAlater^®^ had no impact on protein patterns in skeletal muscle of gulf killifish compared with the snap-freezing method, and suggests we can extend this finding to the mammalian muscle context. 

In relation to tenderness, no significant differences in protein optical density were identified between tender and tough samples, either on day 0 or at day 14 *post mortem* (Table 1). However, highly significant differences were observed for band 7 (*p* = 0.01) and band 9 (*p* < 0.001), between day 0 and day 14 (marked bold in Table 1). As shown in Table 2, there was a decrease in intensity of band 7 and an increase in intensity in band 9, from day 0 to day 14 *post mortem*. 

As bands 7 and 9 showed significant differences during *post mortem* aging, they were further analysed by Liquid chromatography-tandem mass spectrometry. Bands 11 (*p* = 0.08) and 5 (*p* = 0.08) tended towards a treatment effect and an interaction effect of quality × treatment, respectively, so were also analysed by LC-MS/MS. A total of 10 proteins were identified in the four selected bands. Many were enzymes of carbohydrate metabolism, while some were structural proteins (Table S1). Troponin and glyceraldehyde-3-phosphate dehydrogenase were both found in both band 7 and 9 (Table S1). 

The increase in density of band 9 (~30 kDa), from day 0 to day 14, could be explained partly by the appearance of polypeptides migrating at approximately 30 kDa during meat aging, which is in accordance with previous studies of meat tenderization [13,14]. MS of band 9 identified troponin-T, suggesting that this protein may be a proteolyzed breakdown product from band 7. Therefore, we compared the sequence of the observed troponin peptides between band 7 and band 9, results suggested that the KPLN IDHLSEDKLR sequence was not present in band 9 (Figure S1). These findings point to the increased degradation of troponin-T with increasing *post mortem* aging time, which is consistent with reports in the literature [15]. 

The ability to observe proteolytic abundance changes between these two bands has important implications for deducing the relationship between proteolysis of troponin-T and the emergence of a 30 kDa protein post-mortem. It has previously been shown that purified bovine troponin-T can be degraded by µ-calpain in vitro to produce polypeptides in the 30 kDa region [16]. In addition, 30 kDa products of troponin-T have been previously identified by Western blotting [15]. Interestingly, a noteworthy observation to emerge from the data comparison was that samples stored in RNAlater^®^ retained a similar protein profile to samples preserved in dry ice in band 7 and band 9 during the aging time. Taken together, these results show that bovine muscle proteins preserved in RNAlater^®^ present a consistent pattern with those frozen on dry ice and stored at −80 °C, when studied using proteomic approaches.

## 4. Conclusions

This study demonstrates that, based on SDS-PAGE and MS, RNAlater^®^ is a reliable storage agent for bovine muscle tissue that preserves proteins for proteomic analysis in a similar way to freezing in dry ice. It is concluded that use of RNAlater^®^ is suitable for meat proteomic experiments where snap-freezing may not be a viable option for sample stabilization. Further research could focus on different tissue types or other red meat species to establish the wider relevance of our results.

## Figures and Tables

**Table 1:** The *p*-values for effects of quality (tender/tough), treatment (dry ice/RNAlater^®^) and time point (day 0/14) on protein band abundance, and their two-way interactions.

Bands No.	Quality	Treatment	Time Point	Quality × Treatment	Quality × Time Point	Treat × Time Point
1	0.93	0.90	0.17	0.52	0.50	0.94
2	0.58	0.25	0.42	0.57	0.67	0.60
3	0.51	0.51	0.52	0.22	0.61	0.24
4	0.91	0.78	0.93	0.69	0.77	0.71
5	0.34	0.86	0.15	0.08 ^Ϯ^	0.81	0.91
6	0.51	0.83	0.87	0.13	0.74	0.87
7	0.74	0.23	0.01	0.43	0.31	0.39
8	0.98	0.12	0.20	0.94	0.72	0.63
9	0.30	0.33	<001	0.46	0.38	0.88
10	0.87	0.81	0.61	0.84	0.21	0.79
11	0.67	0.08 ^Ϯ^	0.69	0.87	0.71	0.77
12	0.83	0.61	0.86	0.91	0.62	0.54
13	0.64	0.60	0.45	0.45	0.68	0.68
14	0.63	0.21	0.66	0.78	0.96	0.88

*p*-values in bold are significant at the 0.05 level; ^Ϯ^ denotes a tendency with *p* < 0.1.

**Table 2:** Mean protein abundance value of band 7 and band 9.

Dry Ice							RNAlater^®^					
	Day 0	Day 14	e.s.e.	Tender	Tough	e.s.e.	Day 0	Day 14	e.s.e.	Tender	Tough	e.s.e.
Band 7	0.076 ab	0.052 a	0.008	0.066	0.061	0.008	0.101 b	0.056 ab	0.014	0.072	0.085	0.014
Band 9	0.005 a	0.021 b	0.002	0.012	0.015	0.002	0.003 a	0.020 b	0.002	0.011	0.012	0.002

Values within a row that do not share a common superscript differ significantly from each other at the 0.05 level; e.s.e—standard errors of means.

## Supplementary Materials

The following are available online at http://www.mdpi.com/2304-8158/8/2/60/s1, Table S1: Protein identifications from band 5, 7, 9, 11 of SDS-PAGE Gel by LC-MS, Figure S1: Amino acid sequences of peptides of Troponin T in band 7 and 9 identified by mass spectrometry are underlined. The peptide sequence KPLNIDHLSEDKLR (196-210) was detected only in band 7.
