# Supplementary material for: Assessment of RNAlater® as a Potential Method to Preserve Bovine Muscle Proteins Compared with Dry Ice in a Proteomic Study
Source: Foods. 2019 Feb 5;8(2):60. doi: 10.3390/foods8020060 (PMC6406653; doi:10.3390/foods8020060)
Supplement: Supplementary file 1 [file foods-08-00060-s001.pdf]

Supplementary Materials:

1 MSDEEVEHVE EEYEEEEEAQ EEAPPPPAEV PEVHEEVHEV HEPEEVQEEE

51 KPRPRLTAPK IPEGEKVDFD DIQKKRQNKD LMELQALIDS HFEARKKEEE

101ELVALKERIE KRRAERAEQQ RIRAEKERER QNRLAEEKAR REEEDAKRRA

151EDDLKKKKAL SSMGANYSSY LAKADQKRGK KQTAREMKKK VLAERRKPLN

201IDHLSEDKLR DKAKELWDTL YQLETDKFEY GEKLKRQKYD ITNLRSRIDQ

251AQKHSKKAGT APKGKVGGRW K

Figure S1. Amino acid sequences of peptides of Troponin T in band 7 and 9 identified by mass spectrometry are underlined. The peptide sequence KPLNIDHLSEDKLR (196-210) was detected only in band 7.

Table S1. Protein identifications from band 5, 7, 9, 11 of SDS-PAGE Gel by LC-MS.

Identified proteins	Band no.	Accession number (source)	Matched peptides	Theoretical PI	MW (Da) experimental/theoretical	Score	Coverage %
Beta-enolase	5	Q3ZC09 ENOB_BOVIN	31/4	7.60	47409/47096	1120	45
Actin, alpha skeletal muscle	5	P68138 ACTS_BOVIN	16	5.23	42366/42051	672	37
Alpha-enolase	5	P51913 ENOA_CHICK	13	6.17	47617/47305	518	19
Glyceraldehyde-3-phosphate dehydrogenase	7/9	P10096 G3P_BOVIN	26/5	8.51	36073/35868	839/307	46/20
Tropomyosin beta chain	7	Q5KR48 TPM2_BOVIN	7	4.66	32931/32837	514	22
Troponin T, fast skeletal muscle	7/9	Q8MKI3 TNNT3_BOVIN	7/7	5.99	32107/32126	271/265	23/18
Creatine kinase M-type	9	Q9XSC6 KCRM_BOVIN	7	6.63	43190/42989	454	20
Elongation factor Tu	11	A8AWA0 EFTU_STRGC	13	4.86	43983/44011	645	28
Enolase	11	A8AY46 ENO_STRGC	5	4.71	47147/47062	392	18
Trypsin	11	P00761 TRYP_PIG	2	7.00	25078/24409	148	17
